# Visualizing the distribution of black carbon's electron storage capacity using silver

**DOI:** 10.1016/j.mex.2020.100838

**Published:** 2020-02-22

**Authors:** Danhui Xin, Pei C. Chiu

**Affiliations:** Department of Civil and Environmental Engineering, University of Delaware, Newark, DE 19716, United States

**Keywords:** Black carbon, Biochar, Electron storage capacity, Electron donating capacity, Electron accepting capacity, Silver nanoparticles, Electron microscopy, Visualization, Spatial distribution

## Abstract

We have developed a method that combines chemical reduction, silver tagging, and electron microscopy (EM) for visualizing the electron storage capacity (ESC) of black carbon (BC). ESC is a BC's capacity to store and reversibly exchange electrons with abiotic and microbial agents, processes that are relevant to biochemistry, greenhouse gas production, contaminant fate, and remediation. In addition to the amount of electrons BC can store, the locations and spatial distribution of ESC on and inside biochar are critical for understanding the bioaccessibility of ESC and the kinetics of redox reactions involving BC. To locate the ESC in a BC particle, we fully reduced a BC, removed excess reductant, and applied silver ion (Ag^+^) as a tagging agent that diffused into BC to react with functional groups where electrons were stored (i.e., ESC) to form silver nanoparticles (nAg). The nAg deposited on and inside BC were then imaged using multiple EM techniques to visualize the locations and distribution of the ESC. The method is a new and potentially useful tool for investigating ESC production and for elucidating BC-mediated redox transformation.

• Novel method to probe and assess the distribution of ESC on/within BC.
• Visual confirmation of significant ESC both on the surface and in the interior of BC.
• A new method to incorporate silver or other redox-sensitive elements into a carbon medium.

## Graphical abstract

**Figure:**
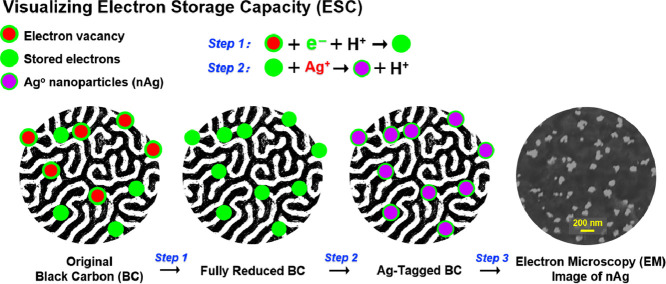

**Table:** Specification Table

Subject Area:	Environmental Science
More specific subject area:	Environmental chemistry
Method name:	Chemical methods combined with electron microscopy
Name and reference of original method:	NA
Resource availability:	

## Method details

### Experimental procedure

A step-by-step procedure is provided for filling, tagging, and visualizing the electron storage capacity (ESC) of black carbon (BC) through the formation of elemental silver nanoparticles (nAg). Step 1 and Step 2 should be conducted in an anaerobic glove box with dissolved O_2_ (DO)-free media to prevent loss of electrons (i.e., oxidation of reduced BC samples).

**Step 1: Reduction of BC (Filling ESC with electrons)**

The first step is to fully reduce redox-active sites, i.e., ESC, using a reducing agent that is (1) strongly reducing and (2) negatively charged and thus would sorb minimally to BC. The reducing agent preferably only serves to transfer electrons to BC. Suitable reducing agents may include Ti(III) citrate and dithionite, the electron content of which can be quantified following the methods recently reported [1].

BC sample is added to a solution containing a pre-determined amount of reducing agent and is allowed to react until the solution's electron content became constant (i.e., stopped decreasing). The electron content of the reducing agent shall be in excess relative to the ESC of the BC sample. After reduction, which may take hours to days depending on the particle size and other properties of the BC, the sample is thoroughly rinsed with DO-free deionized (DI) water, vacuum-dried, and stored in an anaerobic glove box.

A few adjustments can be made in Step 1:

• BC can be drained of electrons through prolonged exposure to DO prior to reduction. The pre-condition through oxidation with DO would be useful for quantifying ESC and establishing electron balance.
• Note that a thoroughly reduced BC sample is not required for silver tagging and visualization (i.e., Steps 2 and 3). In theory, the method can be applied as long as a BC sample contains electrons at a reduction potential favorable for Ag^+^ reduction.

**Step 2: Silver tagging to locate the ESC**

Ag^+^/Ag^o^ has a standard reduction potential (*E*_H_^0^) of +0.80 V vs. standard hydrogen electrode (SHE), which is similar to that of DO at pH 7 (*P*_O2_ 0.21 atm) and higher than the reported *E*_H_^0^ of quinones at pH 7 [2]. Given the ESC of BC was attributed to quinoic moieties [3,4], it would be thermodynamically favorable for Ag^+^ to accept one electron from reduced BC to form Ag^o^. Therefore, when Ag^+^ diffuses into BC and reacts with reduced redox-active moieties such as hydroquinones in the pores, nAg will be formed in situ at or near where the electrons resided. Aqueous Ag^+^ concentration can be monitored to obtain Ag uptake by BC through reduction as well as adsorption. Since adsorbed Ag^+^ would not yield Ag^o^ nanoparticles, only ESC is tagged by nAg. While various silver salts can be used, precipitation and complexation should be considered. For the same reason, pH should be controlled by either a pH controller or a buffer.

**Step 3: Visualization by electron microscopy (EM)**

The difference in atomic weight between Ag and carbon enables visualization of nAg on BC through multiple EM techniques. Scanning electron microscopy (SEM), transmission electron microscopy (TEM), and scanning TEM (STEM) can be used to image the nAg formed on BC surface. To further visualize the spatial distribution of ESC in the interior of BC, EM tomography and EM on a cross-section of BC were performed. The size, density, and distribution of nAg on the surface and in the interior would provide information about the spatial distribution of the ESC.

### Method validation

#### Chemicals

Silver nitrate (99.9+%) and sodium dithionite (>85%) were purchased from Alfa Aesar (Haverhill, MA). Sodium citrate (99%) was acquired from ACROS Organics (Morris Plains, NJ). Sodium nitrate (99.8%), sodium hydroxide solution (1 N, 99.5+%), and nitric acid (67–70%, trace metal grade) were obtained from Fisher Scientific (Hampton, NH). All materials were used as received.

### Examples

BC reduction and Ag tagging experiments were performed in an anaerobic glove box (2.0 ± 0.5% H_2_, 98±0.5% N_2_, P_O2_<25 ppm, Coy, Grass Lake, MI).

**Example 1: Biochar**

SRB [1,^4^,5], a wood-derived biochar, with the size range of 250–500 µm was used as model biochar for method validation.

• Step 1: SRB was first oxidized in air-saturated DI water for 72 h to thoroughly deplete stored electrons. The oxidized SRB was then fully reduced in a solution containing 25 mM dithionite and 0.1 M citrate at pH 6.4 (Measured *E*_H_ = –0.43 V vs. SHE) for 72 h. Due to its low stability, dithionite was added in excess and was replenished as needed to ensure complete reduction of SRB. The reduced SRB was collected on a glass fiber filter disc, rinsed thoroughly with deoxygenated DI water to remove residual reductant and other chemicals, and vacuum-dried prior to Ag^+^ addition.
• Step 2: A known mass (~1 g) of the reduced SRB was suspended in 0.2 L of 0.1 M NaNO_3_ solution in a 1 L amber glass bottle on an orbital shaker at 100 rpm. After equilibration for 30 min with NaNO_3_ solution, a pre-determined aliquot of 100 mM AgNO_3_ was added. Aqueous Ag^+^ concentration was monitored continuously using an Ag^+^ ion selective electrode (ISE) and an ISE meter (Cole–Parmer, Vernon Hills, IL). As Ag^+^ concentration dropped below 1 mM, another aliquot of AgNO_3_ would be added. An automatic pH controller (Bluelab, Tauranga, New Zealand) was used to maintain the solution pH at 7.0 ± 0.4 throughout the experiment using 5 mM NaOH in 0.1 M NaNO_3_. The Ag^+^ ISE was calibrated periodically against Ag^+^ standards. The Ag loading of SRB was calculated from mass balance using Eq. (1).
  (1) $$\text{Ag}\;\text{loading}\;\left(\text{mmol}\right)=C_{{\text{AgNO}}_{3}}\times jV_{{\text{AgNO}}_{3}}-C_{i}\times \left(V_{{\text{NaNO}}_{3}}+jV_{{\text{AgNO}}_{3}}+V_{\text{NaOH}}\right)$$

where *C*_AgNO_3__ and *C_i_* are concentrations of Ag^+^ in the stock solution (100 mM) and in reactor at the time of the *ith* measurement, respectively; *j* is the total number of AgNO_3_ aliquots added; *V*_NaNO_3__ is the initial solution volume (0.2 L), *V*_AgNO_3__ is the volume of AgNO_3_ added each time (0.005 L), and *V_NaOH_* is the total volume of NaOH added.

• Step 3: SEM images were taken for SRB and Ag-tagged SRB using an Auriga 60 CrossBeam high-resolution SEM (ZEISS, Oberkochen, Germany) equipped with a field emission gun (under a vacuum level of 10^−6^–10^−5^ Pa), operating at 1.5–3.0 kV and imaging with secondary electron detectors. For TEM and STEM imaging, Ag-tagged SRB was dispersed in an ethanol solution before transfer to a copper grid with a carbon support film. To obtain the distribution of nAg in the interior of Ag-tagged SRB, a few Ag-tagged SRB particles were cured with low-viscosity Spurr embedding resin at 70 °C in a vacuum oven and sectioned with a diamond knife microtome into 80 nm sections. TEM and STEM images were obtained for the microtomed Ag-tagged SRB using a JEM–2010F (JEOL, Tokyo, Japan) and a Talos F200C (Thermo Fisher Scientific, Waltham, MA) operated at 200 kV. The STEM images were taken using a high-angle annular dark-field (HAADF) detector. Additionally, HAADF–STEM tomography was performed to construct a 3D image of Ag-tagged SRB using a Talos F200C with Fischione tomography sample holder. The sample holder was tilted from –70° to +70° and images were captured every few degrees. Tomography reconstruction was conducted with Avizo and Inspect 3D software (Thermo Fisher Scientific).

**Example 2: Granular activated carbon (GAC)**

OLC, a coconut shell-derived activated carbon, in the size range of 300–500 µm, was used as a model activated carbon.

• Step 1 and Step 2 are the same as for SRB described above.
• Step 3: SEM images were taken for OLC and Ag-tagged OLC.

## Results

**Fig. 1:**
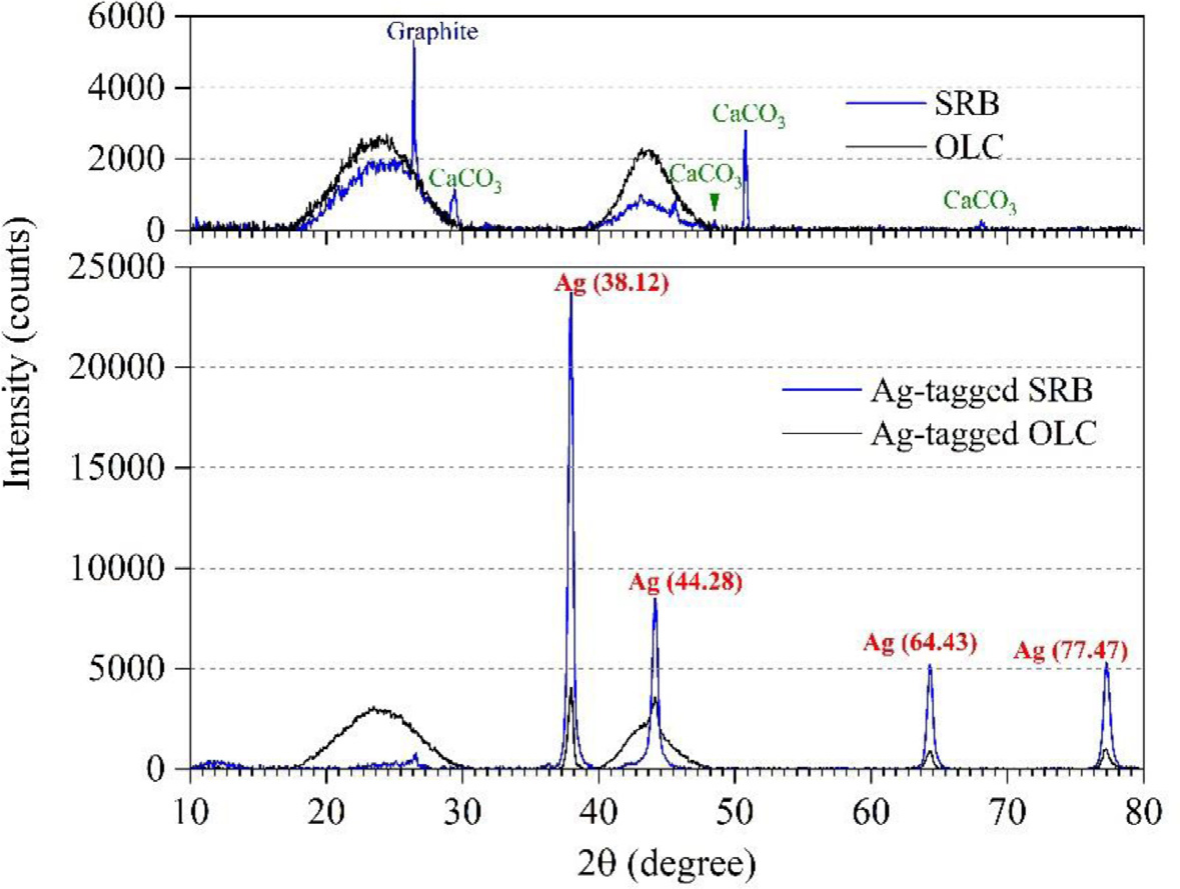
XRD patterns of SRB, OLC, Ag-tagged SRB, and Ag-tagged OLC samples.

The maximum Ag loadings of pre-reduced SRB and OLC at pH 7 were 2.29 mmol/g and 0.99 mmol/g, respectively (Table 1). The formation of elemental Ag^o^ on Ag-tagged BC was verified by the prominent Ag^o^ peaks in X-ray diffraction (XRD) patterns (Fig. 1), whereas none of the Ag^o^ peaks were observed for the original SRB and OLC. The XRD results confirm that Ag^+^ was reductively deposited as Ag^o^ through the ESC. The lower Ag loadings than the ESCs measured with DO for both SRB and OLC might be due to pore blocking by nAg formation/deposition, preventing access of Ag^+^ to the ESC in deeper regions. We note that a higher Ag loading of 2.97 mmol/g for SRB was achievable at pH 8.

**Table 1:** The ESC and Ag loading of BC at pH 7.

	SRB	OLC
ESC (mmol/g)	4.03^a^	2.73^b^
Ag loading (mmol/g)	2.29	0.99

a Reversible ESC between dithionite and DO.

b Reversible ESC between Ti(III) citrate and DO.

**Fig. 2:**
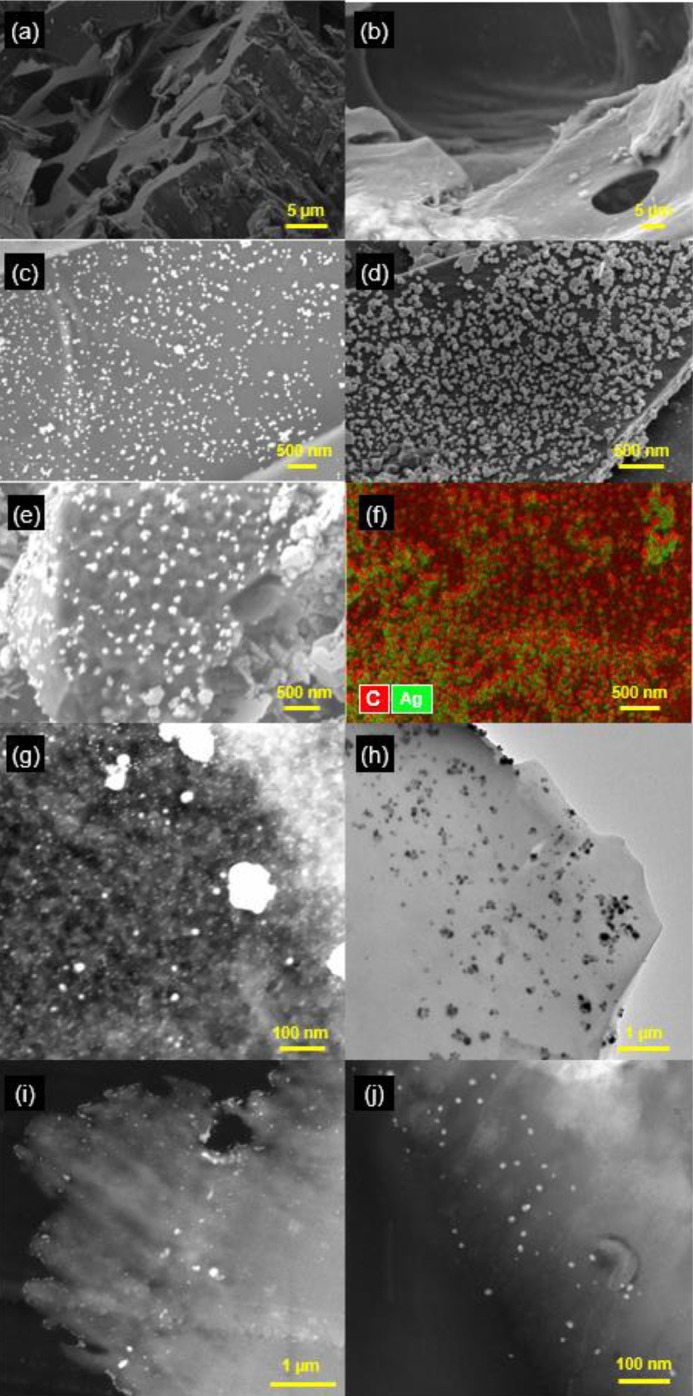
SEM images of (a) and (b) SRB and (c)–(e) Ag-tagged SRB. (f) Elemental mapping of Ag and C on Ag-tagged SRB by SEM–EDS. (g) HAADF–STEM image of Ag-tagged SRB. (h) and (i) TEM and HAADF–STEM images of the microtomed cross-section of a single Ag-tagged SRB particle, respectively. (j) HAADF–STEM image of a cross-section containing small-size (1–10 nm) nAg at high magnification.

**Fig. 3:**
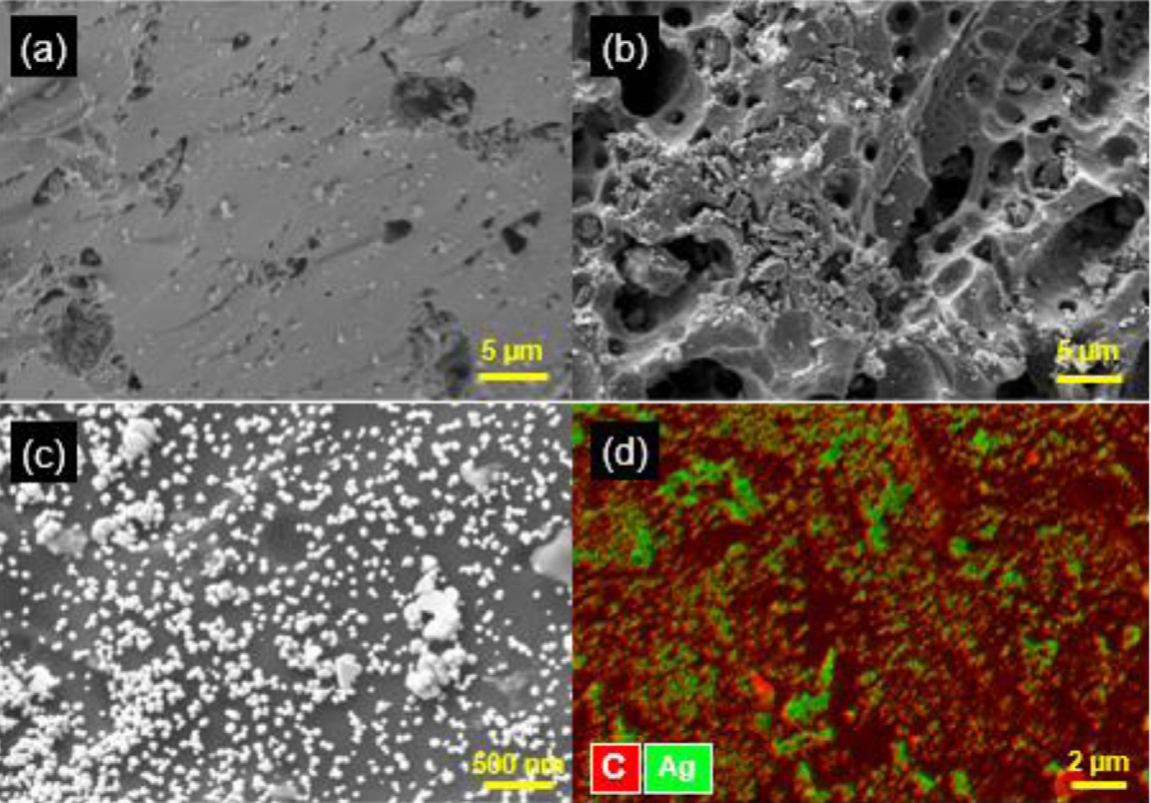
SEM images of (a) and (b) OLC and (c) Ag-tagged OLC. (d) Elemental mapping of Ag and C on Ag-tagged OLC by SEM–EDS.

As shown in Figs. 2(a) and (b), the exterior surface of the SRB without Ag tagging was relatively smooth and few visible nAg were present under EM. In contrast, the surface of Ag-tagged SRB was covered with a large number of nAg (Figs. 2(c)–(e) and (g)) with size ranging from 1 to 100 nm. Similarly, Figs. 3(a) and (b) shows no discernible nAg on the original OLC, whereas nAg were distributed densely and evenly on the surface of Ag-tagged OLC (Fig. 3(c)). In addition, the abundance of Ag on the surface of the Ag-tagged BC was characterized by energy dispersive X-ray spectroscopy (EDS) (Figs. 2(f) and 3(d)). This confirms that using silver tagging and EM techniques, the areal distribution and density of nAg (i.e., ESC) on the surface of BC can be visualized. nAg were observed not only on the outer surface (Fig. 2(g)) but also throughout the interior of SRB when an Ag-tagged SRB particle was microtomed and the cross-section was imaged (Figs. 2(h)–(j)). The fact that nAg existed in the interior of Ag-tagged SRB is consistent with our hypothesis that Ag was incorporated through pore diffusion of aqueous Ag^+^ followed by its reductive deposition within BC. STEM images of the cross-section (Figs. 2(i) and (j)) and a movie visualizing nAg distribution of Ag-tagged SRB (Movie S1, Supplementary data) illustrate that nAg are indeed embedded in the carbon matrix.

### Merits and limits

Mediated electrochemical [3] and chemical [1,4] methods with anionic oxidizing/reducing agents are the established methods for quantifying the ESC of BC. While the Ag tagging method did not capture the full ESC (~57% for SRB, Table 1), it provides a simple way of "seeing" the locations and distribution of ESC. The method thus complements other ESC characterization techniques and can serve as a useful tool for investigating the formation and utilization of black carbon ESC in chemical and microbial studies. In addition to biochar and activated carbon, the method may be applied to other black carbons, including hydrochars, pyrolyzed hydrochars, graphite, (reduced) graphene oxides, soot, and charcoal, with slight modifications.

## Appendix Supplementary materials

Image, video 1
